# Mechanoethology: The Physical Mechanisms of Behavior

**DOI:** 10.1093/icb/icab133

**Published:** 2021-06-14

**Authors:** P A Green, M J McHenry, A Rico-Guevara

**Affiliations:** Centre for Ecology and Conservation, College of Life and Environmental Sciences, University of Exeter, Penryn TR10 9FE, UK; Department of Ecology and Evolutionary Biology, University of California, Irvine, CA 92697, USA; Department of Biology, University of Washington, Seattle, WA 98105, USA; Burke Museum of Natural History and Culture, University of Washington, Seattle, WA 98105, USA

## Abstract

Research that integrates animal behavior theory with mechanics—including biomechanics, physiology, and functional morphology—can reveal how organisms accomplish tasks crucial to their fitness. Despite the insights that can be gained from this interdisciplinary approach, biomechanics commonly neglects a behavioral context and behavioral research generally does not consider mechanics. Here, we aim to encourage the study of “mechanoethology,” an area of investigation intended to encompass integrative studies of mechanics and behavior. Using examples from the literature, including papers in this issue, we show how these fields can influence each other in three ways: (1) the energy required to execute behaviors is driven by the kinematics of movement, and mechanistic studies of movement can benefit from consideration of its behavioral context; (2) mechanics sets physical limits on what behaviors organisms execute, while behavior influences ecological and evolutionary limits on mechanical systems; and (3) sensory behavior is underlain by the mechanics of sensory structures, and sensory systems guide whole-organism movement. These core concepts offer a foundation for mechanoethology research. However, future studies focused on merging behavior and mechanics may reveal other ways by which these fields are linked, leading to further insights in integrative organismal biology.

## Introduction

A central question in organismal biology is: how do organisms accomplish the tasks they face? How does a predatory fish successfully capture evasive prey, and how does the prey avoid capture? How does a male bird defend its territory from conspecific competitors and, later, attract a female mate? One way to answer this central question involves linking behavior—describing the tasks organisms face—with its underlying physical mechanisms, or mechanics—quantifying the movements and forces organisms, and their environments, exert when executing tasks.

The fields of behavior and mechanics have been connected, explicitly or implicitly, for decades. For example, Tinbergen, in his seminal “Four Questions” paper (1963), equated behavior with movement when he defined Ethology as “… ‘the biological study of behaviour’ … characterized by an observable phenomenon (behaviour, or movement).…” From the mechanics side, Koehl (1996) defined biomechanics as the application of engineering techniques to understand how organisms perform functions, including movement. These examples and others (e.g., Garland and Losos 1994; Lauder 1995; Lailvaux and Husak 2014) make clear the importance of integrating research in mechanics with research in behavior. However, despite repeated calls for the broader use of this cross-disciplinary approach (e.g., Byers et al. 2010; Briffa and Lane 2017; Rico-Guevara and Hume 2019), studies of the mechanics of behavior are rare. This may be because, for example, behaviorists have little background with the tools of mechanics research, while those studying mechanics are inexperienced in linking their findings with broader behavioral theories or principles (Losos et al. 2002; Careau and Garland 2012).

Here, we introduce “mechanoethology” as a term intended to encompass research integrating behavior and mechanics. As we detail in examples below, a mechanoethology approach uses techniques from mechanics research (e.g., biomechanics, physiology, functional morphology) to address questions related to, or inspired by, behavioral theory (e.g., sexual selection, collective behavior). Therefore, in addressing the question of how organisms accomplish the tasks they face, mechanoethology research integrates the behavioral theory of *why* organisms undertake those tasks with the biomechanical, physiological, and morphological mechanisms that describe *how* organisms execute those tasks.

This framework is distinct from similar perspectives in its scope of both behavior and mechanics. For example, while Garland and Losos (1994) usefully introduced “behavior” into the classic “morphology → performance → fitness” framework of Arnold (1983), they used behavior as a way to capture how an organism’s performance (usually measured as a maximum value in the lab) is realized in natural interactions (similar to the “realized niche” of Wainwright 1991). Mechanoethology is more specific in suggesting how behavioral *theory* can lead to specific predictions about, among other topics, performance and morphology. These predictions can be tested using mechanics techniques. In a similar vein, the recently introduced field of “mechanical ecology” (Bauer et al. 2020) usefully calls for more field-based approaches to organismal biomechanics. Mechanoethology is distinct in both a broader focus on mechanics writ large (including, e.g., physiology and functional morphology) and a more constrained focus on behavior as opposed to ecology. We note that, while we tend to focus on connections to animal behavior, mechanoethology can extend to nonanimal taxa, including plants (e.g., Crofts and Stankowich 2021).

## Core concepts in mechanoethology

Integrating behavior and mechanics can benefit research in both fields. Table 1 lists core concepts in mechanoethology, showing how research in one field can influence the other. We broadly describe these concepts in this section, referencing later sections in this Introduction and papers in this issue that give more detailed examples.

**Table 1:** Core concepts in mechanoethology. For each concept, we list what insights mechanics can provide behavior and what insights behavior can provide mechanics

Concept	What mechanics provides behavior	What behavior provides mechanics	Examples
Energy	Mechanics sets the energetic cost of motion.	Behavior provides the context and budgeting of energy use.	Schooling fish (Herskin and Steffensen 1998). Hummingbird bills (Rico-Guevara et al. 2021).
Limits	Mechanics imposes physical limits on behavior.	Behavior contributes ecological and evolutionary limits to mechanical systems.	Displaying manakins (Barske et al. 2014). Defensive spines (Crofts and Stankowich 2021).
	Physical limits can be expanded through mechanical innovation.	Limits can be broadened by multifunctionality and behavioral innovation.	“Enhancers,” including springs (Higham and Irschick 2013; Longo et al. 2019). Hummingbird bills (Rico-Guevara and Araya-Salas 2015).
Sensation	Behavioral sensation is underlain by the mechanics of sensory structures.	Sensory behavioral responses form the impetus for and feedback during movement.	Fish predator–prey interactions (Peterson et al. 2021). Competing mantis shrimp (Green et al. 2019; deVries et al. 2021).

Energy is a central currency in both behavior and mechanics. Much animal behavior research is based on building and testing theories related to the costs and benefits of specific movements (Rubenstein and Alcock 2018; Dugatkin and Reeve 2000). While these costs and benefits ultimately affect fitness (e.g., number of surviving offspring), measurements of energy may serve as a proxy for fitness and a link to the mechanics of behavior. The fact that any behavior requires energy, and that energetics is driven by mechanics, means that mechanics sets the energetic costs of behavior. Further, mechanics techniques offer approaches to measuring energy use (e.g., through kinematics or force output). In the “Collective behavior” section, below, we describe how biomechanical studies measuring the energetics of fish swimming help quantify the relative costs and benefits of schooling behavior. From the mechanics perspective, any mechanistic study of the energetics of movement should start by understanding the behavioral context of that movement. If animals are under selection to balance the energetic costs and benefits of their movements (i.e., their behaviors), then mechanistic studies of these movements, and the structures that produce them, are most valid when couched in the appropriate behavioral context. Sargent et al. (2021) contextualize studies on aerodynamics and energetics of hummingbirds with seemingly opposed behavioral strategies, namely stationary interference and traveling exploitation. Also in this lens of mechanoethology, Rico-Guevara et al. (2021) connect hummingbird bill morphology with the energetic costs and benefits of foraging on flowers with different morphologies, all while considering hummingbird behavior, ecology, and evolution.

Mechanics and behavior also provide each other with important limits and, sometimes, ways to bend or break those limits. Any behavior is constrained by its underlying mechanics. For example, force–velocity tradeoffs in muscle physiology mean that muscle contractile force is inherently linked to, and therefore limited by, contractile velocity (Longo et al. 2019). These physical limits mean that some behaviors, no matter how “adaptive” they *could* be, might simply be physically impossible. However, mechanical innovation may provide ways to overcome these limits and expand behavioral boundaries. The section “Animal communication” details studies in golden-collared manakins in which the limits of muscle physiology are stretched, connecting to the evolution of dynamic display behaviors. The limits imposed on mechanics by behavior are not physical, but ecological and/or evolutionary. Any physical mechanism will be influenced, and limited, by the ecology and evolutionary history of the organism(s) involved (e.g., Gould and Lewontin 1979; Wainwright 1991). Ecological and evolutionary boundaries may also be broad, however. For example, some structures are multifunctional, being used for multiple behaviors. This multifunctionality arises because animals evolve new ways of using existing structures (e.g., exaptation, Gould and Vrba 1982). Any study of mechanism should ground itself in the ecological and evolutionary boundaries relevant to that mechanism (and often studied via behavioral approaches). Crofts and Stankowich (2021) describe the ecological and evolutionary forces underlying defensive spine morphology and biomechanics, and the section “Intrasexually selected weaponry” in this Introduction shows how mechanoethology approaches revealed multifunctionality in hummingbird bills (see also Rico-Guevara and Araya-Salas 2015).

Finally, sensation is a key concept in both mechanics and behavior. All organismal behaviors require sensation; for example, a prey cannot avoid a predator without sensing it first. Sensation is inherently a mechanical process, as sensory structures must move to engender a sensory response (e.g., the bending of mechanosensory hairs leads to action potentials). Behavioral studies that consider the mechanics of sensation can find new insights that did not arise without this perspective. The section “Animal communication” and deVries et al. (2021) describe the behavior and mechanics of ritualized striking in mantis shrimp, suggesting how the sensation of strike energy might influence contest behavior. From the mechanics side, any mechanistic study of movement should recognize that the movement came in response to some sensed stimulus (including feedback-based sensation of an organism’s own movement). For example, the movements of both predator and prey in fish predator–prey interactions can be better understood when considering how each party senses the other (Peterson et al. 2021).

## Examples of mechanoethology

In this section, we detail examples from the literature in which mechanoethology approaches have lent insight to both behavior and mechanics research.

### Animal communication

Interacting animals often communicate using signals. For example, male jumping spiders court prospective female mates by displaying color patches (visual signals), waving their legs above their heads (movement-based signals), and drumming their abdomens and legs on the ground (acoustic/vibrational signals) (Echeverri et al. 2017). Female receivers of signals like these are thought to use them to assess a male’s quality and decide whether or not to mate with him (e.g., Brandt et al. 2020). Like those of male jumping spiders, other signals—from acoustic, to chemical, to movement-based—are used by receivers to gather information on the signaler or the environment (Seyfarth et al. 2010). Major questions in animal communication include: what are the mechanics of signal production, and what information do receivers of signals gather from those signals? Mechanoethology approaches have helped develop answers to these questions.

**Fig. 1:**
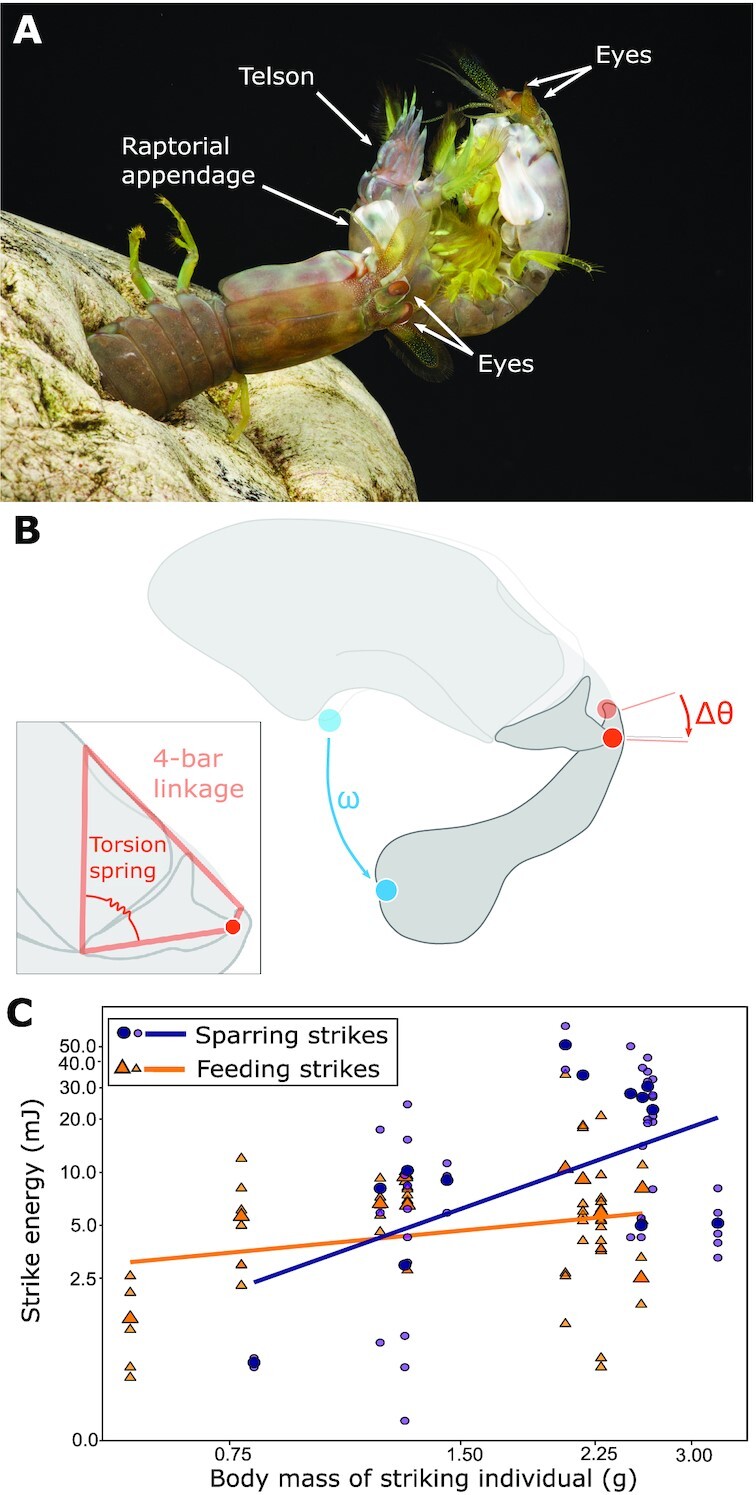
(**A**) A mantis shrimp (left) delivering a raptorial appendage strike onto its competitor’s (right) coiled telson. (**B**) Biomechanical modeling of the strike mechanism showed that the energy used to power a strike can be quantified by measuring strike velocity $\big( {\frac{{\Delta \omega }}{{\mathrm{ time}}}}\big )$. The energy is stored and released from a torsion spring (inset), which is part of a four-bar linkage system (inset) that displaces (θ) to actuate the strike movement. (**C**) Calculating strike energy from the velocity of strikes recorded using high-speed video showed that strike energy increased with increasing body size for sparring strikes (purple), but not feeding strikes (orange). Figures adapted from Green et al. (2019).

Competing individuals are thought to exchange signals that, through energetic costs, help opponents assess their own and/or their competitor’s ability and decide to give up or stay in the fight (reviewed in, e.g., Searcy and Nowicki 2005). Recent work has shown how mechanics approaches can be used to quantify the energetic costs of these signals, helping behaviorists understanding signal assessment. During contests, mantis shrimp (*Neogonodacylus bredini*) ritualistically exchange high-force strikes, delivered by weaponized raptorial appendages onto each other’s armored tailplates in a behavior called “telson sparring” (Green and Patek 2015; Fig. 1A). This sparring serves a communicative function, helping individuals assess relative competitive ability (of which body size is a proxy) and decide to give up a contest instead of simply inflicting damage (Green and Patek 2018). While behaviorists knew that sparring helped competitors gather information about each other, an open question remained: what information is communicated during sparring? That is, what can an individual receiving a strike assess about its competitor? This question was answered with the help of the long history of biomechanics work in mantis shrimp. Earlier studies of mantis shrimp strike biomechanics revealed that an exoskeletal spring stores and releases the energy of the strike (reviewed in Patek 2019). This work led to mathematical models that could quantify the energy required to deliver a strike, given the velocity of the strike and the mass of the striking appendage (McHenry et al. 2012, 2016; Fig. 1B). Green et al. (2019) applied this biomechanics work to quantify the energy of telson sparring strikes. They measured strike velocity from high-speed videos of freely competing mantis shrimp and combined this with measures of competitor appendage mass, incorporating both into the biomechanical model to calculate strike energy. This approach revealed that larger mantis shrimp delivered higher energy sparring strikes than smaller mantis shrimp, a positive scaling not seen when mantis shrimp used their strikes on prey items (snails; Fig. 1C). Because this positive scaling of energy with body size was unique to sparring, and because body size is a proxy of competitive ability (Green and Patek 2018), Green et al (2019) suggested that, during sparring, mantis shrimp strike energy communicates the striking individual’s size and, therefore, its ability. The natural history of mantis shrimp supports this hypothesis. Mantis shrimp live in (and compete over) dark burrows in coral rubble (e.g., Green and Harrison 2020), meaning competitor body size is often hard to assess visually. Sparring might be an efficient means of gathering body size information that would otherwise be hidden. While the physical mechanism by which the receivers of strikes sense strike energy is still unknown, the surfaces of other animal structures used to receive competitive forces have high densities of mechanoreceptors (e.g., stag beetle jaws, Goyens et al. 2015; rhinoceros beetle horns, McCullough and Zinna 2013). Mantis shrimp telsons may similarly have structures that allow for sensation of strike energy (through, e.g., strike force).

Research on mate choice in birds shows how a mechanoethology approach can reveal the energetics of and mechanical limits on animal behavior, as well as how animals stretch mechanical boundaries. Male golden collared manakins produce dynamic signals toward females who are choosing among prospective mates. These signals involve males jumping acrobatically from perch to perch and, while jumping, snapping their wings above their heads to produce sound (a wingsnap; see Clark 2021, this issue, for a discussion of wing-based sound production). Barske et al. (2011) found males that wingsnap more frequently have higher mating success. Further, using high-speed video recordings of signaling behaviors, they found that sub-second differences in male choreography influence female choice. This basic knowledge of the importance of dynamic signaling to mating success led to further questions at the intersection of behavior and mechanics: how costly are these signals, and what physical mechanisms underlie their performance? The first question was answered when Barske et al. (2014) calibrated manakin heart rate with oxygen metabolism-based measures of energy use in the lab. By measuring heart rates of signaling males in the field, the researchers could infer the metabolic costs of signaling from their prior calibration. This clever approach revealed that manakin signals—which increased heart rate to over 1000 bpm—required a remarkable 5.5 kJ/h of energy! This energy expenditure could be prohibitive, but field-based behavioral observations showed that signals last approximately 10 s each, and, on average, manakins signal for only 5 min per day. The result is an average cost of only 1.2% of a manakin’s daily energy budget. Therefore, while signals are instantaneously costly, their long-term costs are quite manageable. Later work using classic muscle physiology approaches revealed that golden collared manakins have evolved superfast muscle contractile physiology to produce such acrobatic signals. Fuxjager et al. (2016) electrically stimulated dissected wing muscles of golden collared manakins, and those of related species, at frequencies similar to those required to produce repeated wingsnaps. By measuring the force outputs of these repeatedly stimulated muscles, they found that the muscle used to produce wingsnaps was able to relax nearly fully, and therefore to remain functional, even over the fast frequencies of stimulation required to produce wingsnaps. In comparison, the same muscles in species that do not produce wingsnaps showed no such ability; neither did other muscles used in flight, but not in signaling, in all species (Fuxjager et al. 2016). This analysis—using techniques most physiologists learn in undergraduate studies—revealed how the limits of muscle force–velocity tradeoffs are stretched in animals that have evolved dynamic, fast signals.

### Intrasexually selected weaponry

The previous examples presented approaches that began with a behavioral observation that led to behavioral hypotheses to be tested through mechanistic approaches. In contrast, in this section we will present a case study that starts with the discovery of sexually dimorphic morphology that did not conform with previous ecological explanations, and the experimental journey to identify its secondary function as an intrasexually selected weapon (ISW). The definition of ISWs, on its own, includes a combination of behavior and morphology linked to a mechanism (Rico‐Guevara and Hurme 2019). A trait can be considered an ISW if it (1) is used as a weapon during same-sex agonistic encounters and (2) is only present or enlarged (relative to body size) in one of the sexes. The concept of ISWs thus goes beyond assumptions of exaggerated sexually dimorphic traits that resemble weapon-like structures but have little behavioral evidence of being used as weapons (e.g., Fig. 4 in Emlen 2008); hence, it is stricter regarding the behavioral evidence of structures actually used as weapons. It also subsumes other widely used terms (e.g., sexually selected weapons, sexually dimorphic weapons, see for instance Rico-Guevara and Araya-Salas 2015) whose definitions do not encompass all ISWs (reviewed in Rico‐Guevara and Hurme 2019). For example, sexually selected weapons are defined as traits used in direct fights between individuals of the same sex over access to individuals of the opposite sex for mating purposes (Berglund 2013); this has as a prerequisite the idea that same-sex fights are over access to mates. The definition of ISWs does not have such prerequisite, making it applicable to cases such as the weaponry of female dung beetles (Emlen et al. 2005), which fight among themselves for dung balls as opposed to battling over males. Another advantage of the term ISWs over other related, widely used terms (e.g., sexually selected weapons, sexually dimorphic weapons) is that it explicitly specifies the kind of sexual selection under which the weapon evolved: intrasexual selection. Other sexually dimorphic weapons have evolved through natural selection (e.g., enlarged mandibles in female soldier ants; Molet et al. 2012); still others under different selective pressures related to sexual selection (e.g., weapons used in sexual coercion; Stutt and Siva-Jothy 2001). These, and other examples (Rico‐Guevara and Hurme 2019), show the importance of relying on behavioral approaches instead of inferring a structure’s function based purely on morphological differences between the sexes. Below, we present a case study that uses both behavior and mechanics approaches to test for the existence of ISWs. We conclude the section with a roadmap of the behavioral determinants for the evolution of ISWs.

The long-billed hermit (*Phaethornis longirostris*) is a hummingbird that has a bill shape sexual dimorphism in length and curvature that had previously been linked to differences in floral resource exploitation between males and females (Temeles et al. 2010). However, Rico-Guevara and Araya-Salas (2015) found dagger-like bill tips in males that were challenging to explain under the ecological causation hypothesis (Temeles et al. 2000, 2010). An alternative explanation for sexually dimorphic traits, even when found in feeding structures (see Rico‐Guevara and Hurme 2019), is that they could serve in combat. Rico-Guevara and Araya-Salas (2015) established four predictions that required approaches from both mechanics and behavior to test: (1) the trait is enlarged or only present in one of the sexes (morphology), (2) it appears or enlarges during puberty (development), (3) it is suited to inflict damage during fights (biomechanics), and (4) weaponized males exhibit elevated dominance status (they have a fighting advantage) that is linked to mating success (behavior). The first two predictions would confirm that the trait is a secondary sexual trait (see Venn diagram in Rico‐Guevara and Hurme 2019) and the last two would corroborate the trait as an ISW, providing an explicit hypothesis for its evolution as a weapon. Through a morphological survey using macrophotography on both museum and live specimens, Rico-Guevara and Araya-Salas (2015) found that adult male *P. longirostris* had pointier and elongated (dagger-like) maxillary overhangs that were absent in females and juveniles, supporting the two first predictions outlined above. Then, the authors assessed if male bills were more suitable to inflict damage when compared to female and juvenile bills, which present bill tips similar to the ones of most other hummingbirds (Rico-Guevara and Rubega 2017). Using a setup designed to measure the force required for a bill to perforate an experimental film, it was shown that *P. longirostris* males with enlarged and pointier bill tips had greater puncture capabilities (i.e., required less force to puncture the film), which would potentially confer a fighting advantage (Rico-Guevara and Araya-Salas 2015). Long-billed hermits are lek breeders: lekking males fiercely defend the perches from where they sing to attract females (Stiles and Wolf 1979). Rico-Guevara and Araya-Salas (2015) recorded agonistic behaviors and captured on video how males stabbed each other with their sexually dimorphic bill tips (also found in other spp., Rico-Guevara et al. 2019). In addition, by using territory mapping and mark-recapture techniques in 5 leks and during 4 years, they demonstrated that males with enlarged and pointier bill tips were more successful in defending lek territories. This final link is suggestive of a relationship between increased fitness and developed weaponry; in other lek breeders, males that defend leks more successfully sire most of the next generation (Balmford et al. 1992; Rintamäki et al. 2001; Isvaran 2021). However, further research has shown that other phenotypic traits are also important to consider when studying the success of these lek breeders (e.g., cognition, Araya-Salas et al. 2018).

Using a mechanoethology approach, all four predictions for these bills to be ISWs were fulfilled. This new ISWs perspective provides an alternative explanation to ecological causation for the origins of sexual dimorphism in hummingbird bills that ought to be explored through future comparative and experimental approaches. For example, sexual dimorphism in bill length and curvature has been attributed to intersexual niche partitioning (Temeles et al. 2000, 2010), yet Rico-Guevara and Araya-Salas (2015) also found that both curvature and pointiness partially explained the lower force adult males need for piercing. Male *P. longirostris* have overall longer and straighter bills than females (Stiles and Wolf 1979; Temeles et al. 2010), additionally, juvenile males transitioned from curved to straighter bills and acquired longer bill tips during puberty (Rico-Guevara and Araya-Salas 2015). Elongated structures are mechanically more resistant to buckling, during axial loading, when they are straighter (e.g., Dahlberg 2004). Hence, longer bills can confer an advantage during bill-sparring through increased reach (same rationale of the “pommeling” technique in fencing), and a straighter bill would be able to transmit more force to the tip resulting in elevated potential damage to an opponent. Lastly, Rico-Guevara and Araya-Salas (2015) found that *P. longirostris* males have thicker bills, potentially better at resisting bending forces when stabbing. Salient questions in this regard include: Do ecological causation and the “bills as ISWs hypothesis” predict the same directionality and kind of sexual dimorphism? Could coevolution between bill shape and flower shape boost underlying sexually dimorphic patterns resulting from a different selective force (e.g., favoring bills as ISWs)? Are ISWs in hummingbird bills also favored by natural selection (via interference competition), are they in opposition to it (via trade-offs with nectar intake efficiency), or both?

### Collective behavior

The study of collective animal behavior aims to understand how the interactions among individuals in a group yield emergent properties for the group (Sumpter 2010). In a number of respects, an animal collective offers more than the sum of its parts, with a capacity for memory, responses to stimuli, and cognitive processing that exceed the abilities of an individual (Sosna et al. 2019). Collective behavior emerges from communication between animals and can be influenced by forces transmitted between them. In fish schools, fluid dynamics offer both a source of information transmission (“Sensation” in Table 1) as well as the means for individuals to influence the propulsion generated by conspecifics, with energetic implications (“Energetics” in Table 1). Schooling consequently offers a system for the study of mechanoethology where behavior both affects, and is affected by, mechanics in multiple respects.

It has long been recognized that fish may swim at a reduced energetic cost when moving in a school (Breder 1926, 1965). This idea is most readily supported by measurements of tail-beat frequency. Tail-beat frequency is tightly correlated with swimming speed in solitary fish (Bainbridge 1958) and the energetic cost of swimming varies with the square or cube of tail-beat frequency (Herskin and Steffensen 1998; Steinhausen et al. 2005). It is therefore energetically meaningful that fish in a school can attain the same speed at a lower frequency than when swimming solitary. For example, a tetra (*Hemigrammus bleheri*) swims at a rapid speed (4 body-lengths/s) with a tail-beat frequency of ∼10 Hz in a school, but requires a frequency of ∼17 Hz to attain the same speed when alone (Ashraf et al. 2017). Similar effects have been observed in mullet (*Liza aurata*; Marras et al. 2015), the intermittent swimming of shiners (*Notemigonus crysoleucas*; Fish et al. 1991), and the pectoral-fin swimming of surfperch (*Embiotoca lateralis*; Johansen et al. 2010). Given the nonlinear relationship with energetic cost, these reductions in frequency have the potential to yield disproportionately large energetic savings (Herskin and Steffensen 1998). However, behaviorists have pointed out that these results should be regarded with caution because metabolic rate is also dependent on the stress level of an animal, and schooling fish are less stressed when surrounded by conspecifics (Nadler et al. 2016). Therefore, an understanding of hydrodynamics is necessary to parse the mechanical benefits of this collective behavior from the confounding influence of reduced stress (Li et al. 2020).

**Fig. 2:**
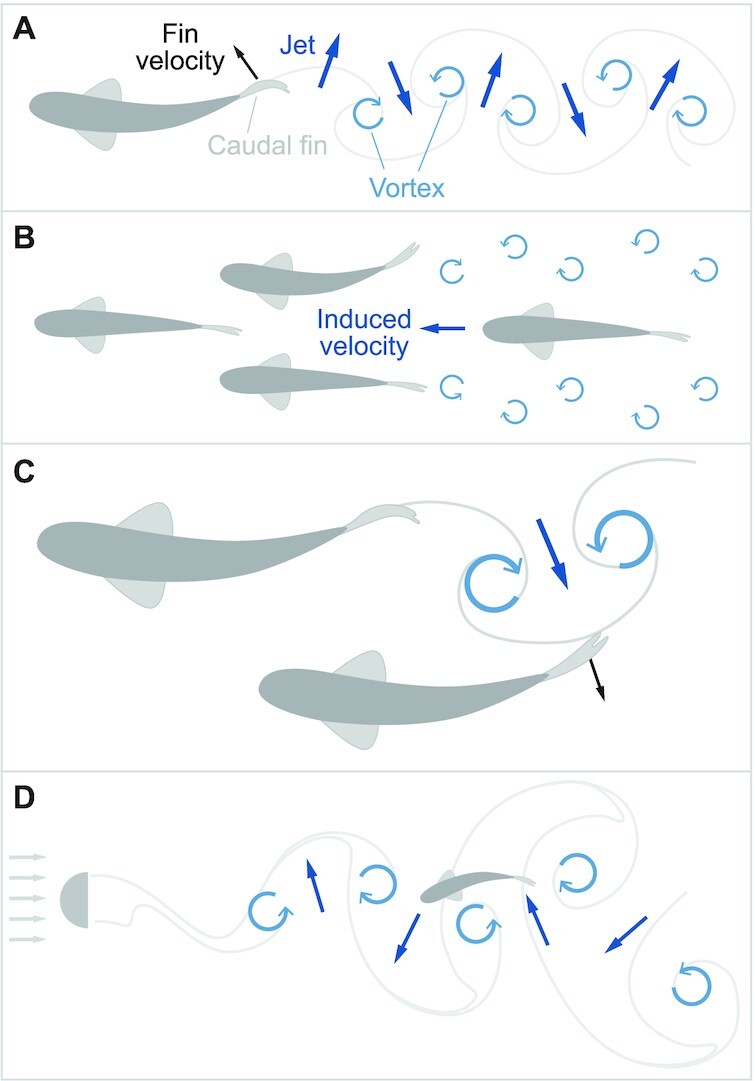
Hydrodynamic mechanisms of enhancing thrust in a wake. (**A**) Thrust generation of a swimming fish includes the shedding of a series of vortices (Rosen 1959). Each vortex features a jet that is directed laterally and in the direction of thrust. (**B**) Weihs (1973) proposed that vortices in the wake between neighboring fish may induce flow in the direction of swimming that could benefit the thrust generated by a trailing fish, a mechanism suggested to work for fish swimming in a diamond-shaped arrangement. (**C**) Vortex phase-matching occurs when a trailing fish coordinates its tail beating relative to the vortices shed by a leading fish to enhance propulsion (Li et al. 2020). (**D**) Trout exhibit a Kármán gait when swimming in the wake of a semicylinder (Liao et al. 2003a). The fish successfully holds station at a relatively low frequency by slaloming between shed vortices.

Hydrodynamic interactions between fish have the potential to enhance thrust production and the efficiency of swimming. Undulatory swimming is characterized by the shedding of a vortex at the lateral excursion of each half tail-beat (Rosen 1959). As a consequence, the wake behind a fish features a series of vortices, each of which resembles a smoke ring of swirling flow with a jet at its center (Fig. 2A). Weihs (1973) suggested that a fish that trails others may benefit by positioning itself between these vortices, where induced flow velocities could be directed in the swimming direction (Fig. 2B). It was predicted that a diamond-shaped arrangement of fish in the school achieves this aim. He additionally proposed that neighboring fish may enhance thrust by channeling flow between their two bodies. In subsequent studies, the diamond pattern has generally not been shown to be exhibited by fish (Partridge and Pitcher 1979; Marras et al. 2015; Ashraf et al. 2017), but it is clear that schooling fish can benefit from hydrodynamic interactions when they swim closely together.

A number of recent studies have offered an explicit consideration of the fluid dynamics of interacting fish bodies, principally through computational fluid dynamics and fish-inspired robots. These approaches consider much of the complexity of deforming bodies in flow that were not possible in Weihs’ (1973) era, including three-dimensional effects, turbulence, and viscous dissipation. This work demonstrates that it is possible for fish to swim more efficiently in a school of seemingly any spatial arrangement. Arrangements in a diamond pattern, aligned in series, side-by-side (i.e., a phalanx), and a square formation can offer energetic savings over solitary swimming (Daghooghi and Borazjani 2015; Hemelrijk et al. 2015; Maertens et al. 2017; Verma et al. 2018; Li et al. 2019a, 2019b; Lin et al. 2019). The manner in which these benefits arise depends on the proximity between the fish; the relative timing of tail beating can also be important. For example, based on measurements from robotic fish, Li et al. (2020) proposed the mechanism of vortex phase-matching. This requires that a follower fish shed vortices with a delay after the leader fish’s such that the jets of the two vortices sum by pointing in a similar direction (Fig. 2C). The tail-beat phase that achieves this depends on the speed of swimming and the relative position of the follower.

A behavioral approach offers the promise of determining whether fish take advantage of the hydrodynamic benefits to schooling. Liao et al. (2003a) made the exciting discovery that trout (*Oncorhynchus mykiss*) adopt a novel gait when holding station in the wake behind a semicylinder (Fig. 2D). This Kármán gait is characterized by swimming at a reduced tail-beat frequency as the fish slaloms between the vortices shed by the cylinder (Liao et al. 2003b). The wake behind a bluff body is different from that of a swimming fish (Fig. 2), but these experiments demonstrate an ability of a fish to position itself and alter its swimming kinematics to take advantage of the vortices within a wake. Based on a kinematic analysis of station-holding by a school, both goldfish (*Carassius auratus*; Li et al. 2020) and tetras (*H. bleheri*; Ashraf et al. 2016) exhibit periods of swimming that are either in-phase with close neighbors, or slightly phase-shifted. However, Li et al. (2020) estimated that goldfish coordinate their swimming with neighbors often enough to realize only about 15% of the total energetic savings possible from vortex phase-matching. Therefore, fishes do coordinate their spacing and timing of swimming to realize energetic gains, but these may not be dominant factors in routine swimming behavior. One might expect energetic economy to be of greatest importance in species that migrate long distances and/or station-hold under intense flow speeds, but it remains to be seen if fishes under these conditions seek energetic savings with greater frequency. Under these conditions, hydrodynamics may offer an explanation for the limits to the speed and energetic costs of swimming in a school (“Limits” in Table 1).

Fluid flow may be sensed by group members, thereby serving as a source of information to facilitate collective behavior. Fishes have a capacity to sense water flow, which can supplement visual cues to regulate the spacing between the members of a collective. The fish lateral line system includes two types of flow receptors. Superficial neuromasts project from the skin, where they encode the velocity of flow at the body’s surface. Canal neuromasts reside within bony canals that have pores at the surface, which allow for the sensing of flow acceleration (van Netten and Kroese 1989; Kroese and Schellart 1992; van Netten and McHenry 2013). Partridge and Pitcher found that individual saithe (i.e., pollock, *Pollachius pollachius*) remain capable of schooling when blinded, but only if the lateral line is fully functional (Pitcher et al. 1976; Pitcher 1979; Partridge and Pitcher 1980). These findings suggest that both the visual and lateral line systems are sufficient for schooling. However, flow sensing has been shown to be insufficient for schooling in a diversity of fishes that fail to maintain a school in the dark, including cyprinids (*Danionella translucida*; Schulze et al. 2018), mackerel (*Trachurus symmetricus*; Hunter 1968), juvenile tuna (*Thunnus orientalis*; Torisawa et al. 2007), juvenile salmon (Ali 2001), and tetras (*Hemigrammus rhodostomus*; McKee et al. 2020). By experimentally compromising the lateral line, a number of studies have demonstrated alterations in the spacing and polarization of schooling fish (e.g., Partridge and Pitcher 1980; Mekdara et al. 2018; McKee et al. 2020). Therefore, the lateral line aids in navigating with respect to neighboring fish, but has largely been shown to be insufficient to maintain a cohesive school.

Birds that fly in formation are potentially capable of realizing energetic benefits to flight in a manner similar to schooling fish. For example, great white pelicans (*Pelecanus onocrotalus*) fly at a reduced heart rate when in formation compared to flying at the same speed on their own (Weimerskirch et al. 2001). A gliding bird, or fixed-wing aircraft, sheds vortices at its wing tip. This swirling flow induces downward flow behind the flier and upward flow (i.e., “upwash”) at positions to the left and right of the wings. Upwash presents an opportunity for a trailing bird to enhance lift generation with flow that is induced by a leading bird, but this requires precise positioning. By GPS tracking individuals, Portugal et al. (2014) demonstrated that northern bald ibises (*Geronticus eremita*) flying in a “V” formation regulate their position to capitalize on the upwash generated by leading birds. In addition, follower birds flap their wings with a delay that follows the wing motion of leaders to track undulations in position of the wing-tip vortices. It is unclear how this coordination is achieved, but bird wings do possess an arrangement of mechanoreceptors that could sense airflow over the wing’s surface (Hörster 1990; Brown and Fedde 1993) and thereby supplement visual cues in a manner analogous to the role played by the lateral line in fishes.

## Conclusion

Integrating behavioral theory with principles and approaches from mechanics can lend new insights to our understanding of how organisms accomplish crucial tasks. We have identified energy, limits, and sensation as key concepts in mechanoethology (Table 1). However, it is likely that there are other ways in which the fields of behavior and mechanics contribute to each other. The other papers in this special issue show how approaches that connect physical mechanisms and behavior can be used to understand topics as diverse as reproduction (Brennan et al. 2021; Johnson et al. 2021), competition (deVries et al. 2021), foraging and avoiding predation (Crofts and Stankowich 2021; Peterson et al. 2021; Rico-Guevara et al. 2021), and communication (Clark 2021). We encourage future studies that further reveal the intersectionality between animal behavior and its underlying mechanics.

## Data Availability

No new data were generated or analyzed in support of this research.

## References

[bib1] Ali M. 2001. The ocular structure, retinomotor and photo-behavioral responses of juvenile pacific salmon. Can J Zool. 37:965–96.

[bib2] Araya-Salas M, Gonzalez-Gomez P, Wojczulanis-Jakubas K, López V, Wright TF. 2018. Spatial memory is as important as weapon and body size for territorial ownership in a lekking hummingbird. Sci Rep. 8:2001. 29386557 10.1038/s41598-018-20441-x PMC5792557

[bib3] Arnold SJ. 1983. Morphology, performance and fitness. Am Zool. 23:347–61.

[bib4] Ashraf I, Bradshaw H, Ha TT, Halloy J, Godoy-Diana R, Thiria B. 2017. Simple phalanx pattern leads to energy saving in cohesive fish schooling. Proc Natl Acad Sci. 114:9599–604. 28839092 10.1073/pnas.1706503114 PMC5594674

[bib5] Ashraf I, Godoy-Diana R, Halloy J, Collignon B, Thiria B. 2016. Synchronization and collective swimming patterns in fish (*Hemigrammus bleheri*). J R Soc Interface. 13:20160734. 27798281 10.1098/rsif.2016.0734 PMC5095228

[bib6] Bainbridge R. 1958. The speed of swimming of fish as related to size and to the frequency and amplitude of the tail beat. J Exp Biol. 35:109–33.

[bib7] Balmford A, Albon S, Blakeman S. 1992. Correlates of male mating success and female choice in a lek-breeding antelope. Behav Ecol. 3:112–23.

[bib8] Barske J, Fusani L, Wikelski M, Feng NY, Santos M, Schlinger BA. 2014. Energetics of the acrobatic courtship in male golden-collared manakins (*Manacus vitellinus*). Proc R Soc B. 281:1–8. 10.1098/rspb.2013.2482 PMC3871312 24352944

[bib9] Barske J, Schlinger BA, Wikelski M, Fusani L. 2011. Female choice for male motor skills. Proc R Soc B. 278:3523–8. 10.1098/rspb.2011.0382 PMC3189371 21508030

[bib10] Bauer U, Poppinga S, Müller UK. 2020. Mechanical ecology—taking biomechanics to the field. Integr Comp Biol. 60:820–8. 32275745 10.1093/icb/icaa018

[bib11] Berglund A. 2013. Why are sexually selected weapons almost absent in females?. Curr Zool. 59:564–8.

[bib12] Brandt EE, Rosenthal MF, Elias DO. 2020. Complex interactions between temperature, sexual signals and mate choice in a desert-dwelling jumping spider. Anim Behav. 170:81–7.

[bib13] Breder CM. 1926. The locomotion of fishes. Zoologica. 4:159–291.

[bib14] Breder CM. 1965. Vortices and fish schools, Zoologica. 50:97–114.

[bib15] Brennan P, Sterett M, DiBuono M, Granados GS, Klo K, Mardsen R, Schleinig T, Tanner L, Purdy S. 2021. Intra-horn penile intromission in the Alpaca *Vicugna pacos* and consequences to genital morphology. Integr Comp Biol. 2. https://doi.org/10.1093/icb/icab050. 10.1093/icb/icab050 33970265

[bib16] Briffa M, Lane SM. 2017. The role of skill in animal contests: a neglected component of fighting ability. Proc R Soc B. 284:20171596. 10.1098/rspb.2017.1596 PMC5627213 28954913

[bib17] Brown RE, Fedde MR. 1993. Airflow sensors in the avian wing. J Exp Biol. 179:13–30.

[bib18] Byers J, Hebets E, Podos J. 2010. Female mate choice based upon male motor performance. Anim Behav. 79:771–8.

[bib19] Careau V, Garland T. 2012. Performance, personality, and energetics: correlation, causation, and mechanism. Physiol Biochem Zool. 85:543–71. 23099454 10.1086/666970

[bib20] Clark C. 2021. Ways that animal wings produce sound. Integr Comp Biol. 2. https://doi.org/10.1093/icb/icab008. 10.1093/icb/icab008 33693721

[bib21] Crofts S, Stankowich T. 2021. Stabbing spines: a review of the biomechanics and evolution of defensive spines in plants and animals. Integr Comp Biol. 2. https://doi.org/10.1093/icb/icab099. 10.1093/icb/icab099 34038530

[bib22] Daghooghi M, Borazjani I. 2015. The hydrodynamic advantages of synchronized swimming in a rectangular pattern. Bioinspir Biomim. 10:056018. 26447493 10.1088/1748-3190/10/5/056018

[bib23] Dahlberg T. 2004. Procedure to calculate deflections of curved beams. Int J Eng Educ. 20:503–13.

[bib24] deVries M, Lowder K, Taylor J. 2021. From telson to attack in mantis shrimp: bridging biomechanics and behavior in crustacean conflicts. Int Comp Biol. 2. https://doi.org/10.1093/icb/icab064. 10.1093/icb/icab064 33974067

[bib25] Dugatkin LA, Reeve HK. 2000. Game theory and animal behavior. Oxford: Oxford University Press.

[bib26] Echeverri SA, Morehouse NI, Zurek DB, Holman L. 2017. Control of signaling alignment during the dynamic courtship display of a jumping spider. Behav Ecol. 28:1445–53.

[bib27] Emlen DJ. 2008. The evolution of animal weapons. Annu Rev Ecol Evol Syst. 39:387–413.

[bib28] Emlen DJ, Marangelo J, Ball B, Cunningham CW. 2005. Diversity in the weapons of sexual selection: horn evolution in the beetle genus *Onthophagus* (Coleoptera: Scarabaeidae). Evolution. 59:1060–84. 16136805

[bib29] Fish FE, Fegely JF, Xanthopoulos CJ. 1991. Bust-and-coast swimming in schooling fish (*Notemigonus crysoleucas*) with implications for energy economy. Comp Biochem Physiol A. 100:633–7.

[bib30] Fuxjager MJ, Goller F, Dirkse A, Sanin GD, Garcia S. 2016. Select forelimb muscles have evolved superfast contractile speed to support acrobatic social displays. Elife. 5:e13544. 27067379 10.7554/eLife.13544 PMC4829423

[bib31] Garland TJ, Losos JB. 1994. Ecological morphology of locomotor performance in squamate reptiles. In: Wainwright PC, Reilly SM, editors. Ecological morphology: integrative organismal biology. Chicago & London: The University of Chicago Press.

[bib32] Gould SJ, Lewontin RC. 1979. The spandrels of San Marco and the Panglossian paradigm: a critique of the adaptationist programme. Proc R Soc B. 205:581–98. 42062 10.1098/rspb.1979.0086

[bib33] Gould SJ, Vrba ES. 1982. Exaptation—a missing term in the science of form. Paleobiology. 8:4–15.

[bib34] Goyens J, Dirckx J, Aerts P. 2015. Mechanoreceptor distribution in stag beetle jaws corresponds to the material stress in fights. Arthropod Struct Dev. 44:201–8. 25843903 10.1016/j.asd.2015.03.003

[bib35] Green PA, Harrison JS. 2020. Quadratic resource value assessment during mantis shrimp (stomatopoda) contests. Anim Behav. 170:207–18.

[bib36] Green PA, McHenry MJ, Patek SN. 2019. Context-dependent scaling of weapon kinematics and energetics in mantis shrimp (Stomatopoda). J Exp Biol. 222:1–12. 10.1242/jeb.198085 30890620

[bib37] Green PA, Patek SN. 2015. Contests with deadly weapons: Telson sparring in mantis shrimp (Stomatopoda). Biol Lett. 11:1–4. 10.1098/rsbl.2015.0558 PMC4614432 26399976

[bib38] Green PA, Patek SN. 2018. Mutual assessment during ritualized fighting in mantis shrimp (Stomatopoda). Proc R Soc B. 285:1–9. 10.1098/rspb.2017.2542 PMC5805947 29343603

[bib39] Hemelrijk C, Reid D, Hildenbrandt H, Padding J. 2015. The increased efficiency of fish swimming in a school. Fish Fisheries. 16:511–21.

[bib40] Herskin J, Steffensen J. 1998. Energy savings in sea bass swimming in a school: measurements of tail beat frequency and oxygen consumption at different swimming speeds. J Fish Biol. 53:366–76.

[bib41] Higham TE, Irschick DJ. 2013. Springs, steroids, and slingshots: the role of enhancers and constraints in animal movement. J Comp Physiol B. 183:583–95. 23292335 10.1007/s00360-012-0734-z

[bib42] Hörster W. 1990. Histological and electrophysiological investigations on the vibration-sensitive receptors (*Herbst corpuscles*) in the wing of the pigeon (*Columba livia*). J Comp Physiol A. 166:663–73.

[bib43] Hunter JR. 1968. Effects of light on schooling and feeding of jack mackerel, *Trachurus symmetricus*. J Fish Res Board Can. 25:393–407.

[bib44] Isvaran K. 2021. Lek territory size and the evolution of leks: a model and a test using an ungulate with a flexible mating system. Front Ecol Evol. 8:1–14.

[bib45] Johansen J, Vaknin R, Steffensen J, Domenici P. 2010. Kinematics and energetic benefits of schooling in the labriform fish, striped surfperch *Embiotoca lateralis*. Mar Ecol Prog Ser. 420:221–9.

[bib46] Johnson MA, Kamath A, Kirby R, Fresquez CC, Wang S, Stehle CM, Templeton AR, Losos J. 2021. What determines paternity in wild lizards? Analyses through territorial and non-territorial lenses. Integr Comp Biol. 2. https://doi.org/10.1093/icb/icab115. 10.1093/icb/icab115 34077526

[bib47] Koehl M. 1996. When does morphology matter?. Annu Rev Ecol Syst. 27:501–42.

[bib48] Kroese A, Schellart N. 1992. Velocity-and acceleration-sensitive units in the trunk lateral line of the trout. J Neurophysiol. 68:2212–21. 1491267 10.1152/jn.1992.68.6.2212

[bib49] Lailvaux SP, Husak JF. 2014. The life history of whole-organism performance. Q Rev Biol. 89:285–318. 25510077 10.1086/678567

[bib50] Lauder GV. 1995. On the inference of function from structure. In: Thomason JJ, editor. Functional morphology in vertebrate paleontology. Cambridge: Cambridge University Press. p. 1–18.

[bib51] Li G, Kolomenskiy D, Liu H, Thiria B, Godoy-Diana R. 2019b. On the energetics and stability of a minimal fish school. PLoS One. 14:e0215265. 31461457 10.1371/journal.pone.0215265 PMC6713342

[bib52] Li L, Nagy M, Graving JM, Bak-Coleman J, Xie G, Couzin ID. 2020. Vortex phase matching as a strategy for schooling in robots and in fish. Nat Commun. 11:1–9. 33106484 10.1038/s41467-020-19086-0 PMC7588453

[bib53] Li S, Li C, Xu L, Yang W, Che X. 2019a. Numerical simulation and analysis of fish-like robots swarm. Appl Sci. 9:1652.

[bib55] Liao JC, Beal DN, Lauder GV, Triantafyllou MS. 2003a. The Kármán gait: novel body kinematics of rainbow trout swimming in a vortex street. J Exp Biol. 206:1059–73. 12582148 10.1242/jeb.00209

[bib54] Liao JC, Beal DN, Lauder GV, Triantafyllou MS. 2003b. Fish exploiting vortices decrease muscle activity. Science. 302:1566–9. 14645849 10.1126/science.1088295

[bib56] Lin X, Wu J, Zhang T, Yang L. 2019. Phase difference effect on collective locomotion of two tandem autopropelled flapping foils. Phys Rev Fluids. 4:054101.

[bib57] Longo SJ, Cox SM, Azizi E, Ilton M, Olberding JP, St Pierre R, Patek SN. 2019. Beyond power amplification: latch-mediated spring actuation is an emerging framework for the study of diverse elastic systems. J Exp Biol. 222:1–10. 10.1242/jeb.197889 31399509

[bib58] Losos JB, Creer DA, Shulte JAI. 2002. Cautionary comments on the measurement of maximum locomotor capabilities. J Zool. 258:57–61.

[bib59] Maertens AP, Gao A, Triantafyllou MS. 2017. Optimal undulatory swimming for a single fish-like body and for a pair of interacting swimmers. J Fluid Mech. 813:301–45.

[bib60] Marras S, Killen SS, Lindström J, McKenzie DJ, Steffensen JF, Domenici P. 2015, Fish swimming in schools save energy regardless of their spatial position, Behav Ecol Sociobiol. 69:219–26. 25620833 10.1007/s00265-014-1834-4 PMC4293471

[bib61] McCullough EL, Zinna RA. 2013. Sensilla density corresponds to the regions of the horn most frequently used during combat in the giant rhinoceros beetle *Trypoxylus dichotomus* (Coleoptera: Scarabaeidae: Dynastinae). Ann Entomol Soc Am. 106:518–23.

[bib62] McHenry MJ, Anderson PS, VanWassenbergh S, Matthews DG, Summers AP, Patek SN. 2016. The comparative hydrodynamics of rapid rotation by predatory appendages. J Exp Biol. 219:3399–411. 27807217 10.1242/jeb.140590

[bib63] McHenry MJ, Claverie T, Rosario MV, Patek SN. 2012. Gearing for speed slows the predatory strike of a mantis shrimp. J Exp Biol. 215:1231–45. 22399669 10.1242/jeb.061465

[bib64] McKee A, Soto AP, Chen P, McHenry MJ. 2020. The sensory basis of schooling by intermittent swimming in the rummy-nose tetra (*Hemigrammus rhodostomus*). Proc Roy Soc B. 287:20200568. 10.1098/rspb.2020.0568 PMC7661294 33109007

[bib65] Mekdara PJ, Schwalbe MAB, Coughlin LL, Tytell ED. 2018. The effects of lateral line ablation and regeneration in schooling giant danios. J Exp Biol. 221:175166. 10.1242/jeb.175166 29530974

[bib66] Molet M, Wheeler DE, Peeters C. 2012. Evolution of novel mosaic castes in ants: modularity, phenotypic plasticity, and colonial buffering. Am Nat. 180:328–41. 22854076 10.1086/667368

[bib67] Nadler LE, Killen SS, McClure EC, Munday PL, McCormick MI. 2016. Shoaling reduces metabolic rate in a gregarious coral reef fish species. J Exp Biol. 219:2802–5. 27655821 10.1242/jeb.139493 PMC5047653

[bib68] Partridge BL, Pitcher TJ. 1980. The sensory basis of fish schools: relative roles of lateral line and vision. J Comp Phys A. 135:315–25.

[bib69] Patek SN. 2019. The power of mantis shrimp strikes: interdisciplinary impacts of an extreme cascade of energy release. Integr Comp Biol. 59:1573–85. 31304967 10.1093/icb/icz127

[bib70] Peterson AN, Soto A, McHenry M. 2021. Pursuit and evasion strategies in the predator-prey interactions of fishes. Integr Comp Biol. 2. https://doi.org/10.1093/icb/icab116. 10.1093/icb/icab116 34061183

[bib71] Pitcher TJ. 1979. Sensory information and the organization of behaviour in a shoaling cyprinid fish. Anim Behav. 27:126–49.

[bib72] Pitcher TJ, Partridge BL, Wardle C. 1976. A blind fish can school. Science. 194, 963–5. 982056 10.1126/science.982056

[bib73] Portugal SJ, Hubel TY, Fritz J, Heese S, Trobe D, Voelkl B, Hailes S, Wilson AM, Usherwood JR. 2014. Upwash exploitation and downwash avoidance by flap phasing in ibis formation flight. Nature. 505:399–402. 24429637 10.1038/nature12939

[bib74] Rico-Guevara A, Araya-Salas M. 2015. Bills as daggers? A test for sexually dimorphic weapons in a lekking hummingbird. Behav Ecol. 26:21–9.

[bib75] Rico-Guevara A, Hurme KJ. 2019. Intrasexually selected weapons. Biol Rev. 94:60–101. 10.1111/brv.12436 29924496

[bib76] Rico-Guevara A, Hurme KJ, Elting R, Russel AL. 2021. Bene‘fit’ assessment in pollination coevolution: mechanistic perspectives on hummingbird bill-flower matching. Integr Comp Biol. 2. https://doi.org/10.1093/icb/icab111. 10.1093/icb/icab111 34050734

[bib77] Rico-Guevara A, Rubega MA. 2017. Functional morphology of hummingbird bill tips: their function as tongue wringers. Zoology. 123:1–10. 28760683 10.1016/j.zool.2017.06.001

[bib78] Rico-Guevara A, Rubega MA, Hurme KJ, Dudley R. 2019. Shifting paradigms in the mechanics of nectar extraction and hummingbird bill morphology. Integr Org Biol. 1:1–15. 10.1093/iob/oby006 PMC7671138 33791513

[bib79] Rintamäki PT, Höglund J, Alatalo RV, Lundberg A. 2001. Correlates of male mating success on black grouse (*Tetrao tetrix* L.) leks. Ann Zool Fenn. 38:99–109.

[bib80] Rosen MW. 1959. Water flow about a swimming fish. US Naval Ordnance Test Station 2298. Los Angeles: University of California in Los Angeles.

[bib81] Rubenstein DR, Alcock J. 2018. Animal behavior. Oxford: Oxford University Press.

[bib82] Sargent AJ, Groom DJE, Rico-Guevara A. 2021. Locomotion and energetics of divergent foraging strategies in hummingbirds: a review. Integr Comp Biol. 2. https://doi.org/10.1093/icb/icab124. 10.1093/icb/icab124 34113992

[bib83] Schulze L, Henninger J, Kadobianskyi M, Chaigne T, Faustino AI, Hakiy N, Albadri S, Schuelke M, Maler L, Del Bene F et al. 2018. Transparent *Danionella translucida* as a genetically tractable vertebrate brain model. Nat Methods. 15:977–83. 30323353 10.1038/s41592-018-0144-6

[bib84] Searcy WA, Nowicki S. 2005. The evolution of animal communication: reliability and deception in signaling systems. Princeton: Princeton University Press.

[bib85] Seyfarth RM, Cheney DL, Bergman T, Fischer J, Zuberbühler K, Hammerschmidt K. 2010. The central importance of information in studies of animal communication. Anim Behav. 80:3–8.

[bib87] Sosna MM, Twomey CR, Bak-Coleman J, Poel W, Daniels BC, Romanczuk P, Couzin ID. 2019. Individual and collective encoding of risk in animal groups. Proc Natl Acad Sci. 116:20556–61. 31548427 10.1073/pnas.1905585116 PMC6789631

[bib88] Steinhausen MF, Steffensen JF, Andersen NG. 2005. Tail beat frequency as a predictor of swimming speed and oxygen consumption of saithe (*Pollachius virens*) and whiting (*Merlangius merlangus*) during forced swimming. Mar Biol. 148:197–204.

[bib89] Stiles FG, Wolf LL. 1979. Ecology and evolution of lek mating behavior in the long-tailed hermit hummingbird. Ornithological monographs. Washington, D.C.: Amer Ornithologists Union. p. iii–78.

[bib90] Stutt AD, Siva-Jothy MT. 2001. Traumatic insemination and sexual conflict in the bed bug *Cimex lectularius*. Proc Natl Acad Sci. 98:5683–7. 11331783 10.1073/pnas.101440698 PMC33273

[bib91] Sumpter DJ. 2010. Collective animal behavior. Princeton, NJ: Princeton University Press.

[bib92] Temeles EJ, Miller JS, Rifkin JL. 2010. Evolution of sexual dimorphism in bill size and shape of hermit hummingbirds (Phaethornithinae): a role for ecological causation. Phil Trans R Soc B: Biol Sci. 365:1053–63. 10.1098/rstb.2009.0284 PMC2830232 20194168

[bib93] Temeles EJ, Pan IL, Brennan JL, Horwitt JN. 2000. Evidence for ecological causation of sexual dimorphism in a hummingbird. Science. 289:441–3. 10903203 10.1126/science.289.5478.441

[bib94] Tinbergen N. 1963. On aims and methods of ethology. Z Für Tierpsychol. 20:410–33.

[bib95] Torisawa S, Takagi T, Fukuda H, Ishibashi Y, Sawada Y, Okada T, Miyashita S, Suzuki K, Yamane T. 2007. Schooling behaviour and retinomotor response of juvenile Pacific bluefin tuna *Thunnus orientalis* under different light intensities. J Fish Biol. 71:411–20.

[bib96] van Netten SM, Kroese A. 1989. Dynamic behavior and micromechanical properties of the cupula. In: Coombs S, Gorner P, Munz H, editors. The mechanosensory lateral line: neurobiology and evolution. Berlin: Springer-Verlag. p. 247–63.

[bib86] van Netten SM, McHenry MJ. 2013. The biophysics of the fish lateral line. In: Coombs S, Bleckmann H, Fay RR, Popper AN, editors. The lateral line. New York: Springer. p. 99–120.

[bib97] Verma S, Novati G, Koumoutsakos P. 2018. Efficient collective swimming by harnessing vortices through deep reinforcement learning. Proc Natl Acad Sci. 115:5849–54. 29784820 10.1073/pnas.1800923115 PMC6003313

[bib98] Wainwright PC. 1991. Ecomorphology: experimental functional anatomy for ecological problems. Am Zool. 31:680–93.

[bib99] Weihs D. 1973. Hydromechanics of fish schooling. Nature. 241:290–1.

[bib100] Weimerskirch H, Martin J, Clerquin Y, Alexandre P, Jiraskova S. 2001. Energy saving in flight formation. Nature. 413:697–8. 11607019 10.1038/35099670

